# Supplementary material for: Prevalence of Salmonella in Free-Range Pigs: Risk Factors and Intestinal Microbiota Composition
Source: Foods. 2021 Jun 18;10(6):1410. doi: 10.3390/foods10061410 (PMC8235412; doi:10.3390/foods10061410)
Supplement: Supplementary file 1 [file foods-10-01410-s001.zip › 2_ Suplementary Tables S1-S2_SENT_R1.pdf]

SUPPLEMENTARY MATERIAL

Prevalence of *Salmonella* in free-range pigs: risk factors and intestinal microbiota composition

Victoria Garrido 1, Lourdes Migura-García 2, Inés Gaitán 1, Ainhoa Arrieta-Gisasola 3, Ilargi Martínez-Ballesteros 3, Lorenzo Fraile 4, and María Jesús Grilló 1,*

[1] Animal Health Group, Instituto de Agrobiotecnología (CSIC-Gobierno de Navarra) Mutilva, Navarra, Spain; victoria.garrido@csic.es; inesgaitanmarqueta@gmail.com; mj.grillo@csic.es

[2] IRTA, Centre de Recerca en Sanitat Animal (CReSA, IRTA-UAB), Universitat Autònoma de Barcelona, Campus UAB, 08193 Bellaterra, Barcelona, Spain; OIE Collaborating Centre for the Research and Control of Emerging and Re-emerging Swine Diseases in Europe (IRTA-CReSA), Bellaterra, Barcelona, Spain; lourdes.migura@irta.cat

[3] MikroIker research group, Immunology, Microbiology and Parasitology Department, Faculty of Pharmacy, University of the Basque Country (UPV/EHU), Vitoria-Gasteiz, Spain; Bioaraba, Vitoria-Gasteiz, Spain; ainhoa.arrieta@ehu.eus; ilargi.martinez@ehu.eus

[4] Departamento de Ciencia Animal, Universidad de Lleida, Lleida, Spain; lorenzo.fraile@udl.cat

* Correspondence: mj.grillo@csic.es

Table S1. Contingency table with results of *Salmonella* identification by ISO 6579:2002/Amend. 1:2007 (ISO) and/or PCR-*invA* using MRSV as DNA source (PCR).

	**ISO +**	**ISO -**	**Totals**
**PCR +**	47	11	58
**PCR -**	0	122	122
**Totals**	47	133	180

Table S2. Classification of farms by *Salmonella* prevalence in the intestinal content of free-range pigs and the risk factors associated by univariable analysis.

**Farms characteristics**			**Risk factors**		
**Farm code**	***Salmonella* prevalence**	**Number of animals/farm (n)**[1]	**Dry feed Diet**	**Extra-Diet**	**Silo´s cleaning and disinfection**
1	86.7%	140 (n=10)	A	Grass	Shallow/No
2	53.3%	130	B	Acorn	Shallow/No
3	46.7%	160 (n=5)	A	Grass	Shallow/No
4	46.7%	140	A	Grass	Shallow/No
5	40%	110	A	Acorn	Shallow/No
6	33.3%	140	A	Grass	Shallow/No
7	26.7%	80	A	Grass	Shallow/No
8	26.7%	120	A	Grass	Shallow/No
9	20%	140	B	Chestnut and Beechnut	Frequent/Yes
10	6.7%	100	B	Acorn	Frequent/Yes
11	0	100 (n=10)	B	Acorn	Frequent/Yes
12	0	100 (n=10)	C	Chestnut and Beechnut	Shallow/No

[1] Farms and number of pigs (n) selected for the microbiome study, showing high (n=15) or null (n=20) *Salmonella* prevalence.
